# Genetics of antibiotic resistance in methicillin-resistant *Staphylococcus aureus* (MRSA)

**DOI:** 10.5114/bta.2024.139756

**Published:** 2024-06-25

**Authors:** Klementyna Marciniak, Agata Tyczewska, Kamilla Grzywacz

**Affiliations:** Institute of Bioorganic Chemistry, Polish Academy of Sciences, Poznań, Poland

**Keywords:** *Staphylococcus aureus*, MRSA, antibiotic resistance

## Abstract

Methicillin-resistant *Staphylococcus aureus* (MRSA) strains pose a significant threat as common causes of bacterial infections in hospitals, often resistant to available antibiotics such as daptomycin, vancomycin, and linezolid. The continuous emergence of new MRSA isolates with no effective treatment options underscores a real threat to health among humans and animals, and the number of effective antibiotic therapies decreases with each passing year. In this review, we provide an overview of the most common genetic mechanisms of resistance to a broad spectrum of antibiotics in methicillin-resistant *S. aureus*.

## Introduction

Throughout evolution, all spreading life forms have developed mechanisms to be as competitive as possible in the race for access to nutrients and territory. Among microorganisms, one of the most effective methods of eliminating competition is the secretion of antibiotics, which exhibit a diverse range of actions that effectively limit the growth and development of specific organisms.

When Alexander Fleming gave a speech upon receiving the Nobel Prize for the discovery of penicillin G, he warned against the hasty and ill-considered use of antibiotics due to the possibility of the emergence and rapid spread of bacterial resistance (Fleming, Nobel Lecture, 1945). The natural response to the action of an inhibitory factor is for microorganisms to develop mechanisms enabling their neutralization. This fact means that after 50 years of widespread antibiotic use, humanity is facing another significant threat.

According to the first global report on antibiotic resistance presented by the World Health Organization (WHO) in 2018, the world is heading towards a postantibiotic era in which common infections and minor injuries that have been effectively treated for decades may again be fatal (World Health Organization, 2018). Each year, this problem affects as many as 700,000 people who die as a result of failed antibiotic treatment. It is estimated that after 2050, this number will increase to 10 million, which currently corresponds to the annual number of deaths due to cancer (World Health Organization, 2024). The search for alternative solutions or new antimicrobial drugs has become a priority but also a huge challenge for scientists. Antimicrobial resistance has been identified as one of the top 10 global public health threats facing humanity (EClinicalMedicine, 2021).

In the Center’s for Disease Control and Prevention (CDC) report *Antibiotic Resistance Threats in the United States, 2019*, regarding the persistent problem of antibiotic resistance in the United States, a methicillinresistant strain of *Staphylococcus aureus*, MRSA, is listed as a serious threat (CDC, 2019). Statistics for this microorganism rank among the most dangerous infections, both in hospitals and communities. In the USA alone, in 2017, 10,600 people died due to MRSA infection, and 323,700 were hospitalized. Treatment costs in the same year exceeded 1.7 billion USD, constituting the largest share of expenditure allocated to the fight against multidrug-resistant infections. The widespread and intensive use of antimicrobials has led to a partial loss of control over verifying the validity of their use. This applies to both primary and hospital health care, as well as veterinary medicine, animal fattening, and plant production. It is believed that the sum of such activities over the last 50 years has exerted significant selective pressure on microbial populations, promoting strains capable of rapid adaptation (Aggrawal et al., 2024). Accordingly, the threat posed by the uncontrolled worsening of the drug resistance crisis has stimulated efforts to gain insight into the genetics and identify the determinants encoding antibiotic resistance. Currently, the name MRSA no longer reflects the true nature of these organisms. This is not only methicillin resistance but also resistance to over 20 other different antimicrobial compounds, including vancomycin, called the drug of last resort (Shah et al., 2024).

## MRSA strains with different susceptibility to vancomycin

In 1996, in Japan, the first clinical isolate of *S. aureus* MRSA, strain Mu50, showing reduced sensitivity to vancomycin, was identified (Hiramatsu et al., 1997). Hence its names: VISA, derived from “vancomycin-intermediate *S. aureus* ”, or GISA, from “glycopeptide intermediate *S. aureus* ” (the latter showed reduced sensitivity to teicoplanin).

Interest in MRSA was aroused with the emergence of subpopulations with different minimum inhibitory concentrations (MICs) of vancomycin. The minimum inhibitory concentration is defined as the lowest concentration of an antimicrobial agent at which inhibition of microbial growth is observed. Due to documented cases of patients from all over the world in whom vancomycin therapy was unsuccessful, doctors and microbiologists reverified the MIC values for this antibiotic (Cui et al., 2000). According to statistics, if this value exceeded 2 mg/l, the treatment was significantly difficult or ineffective. For this reason, in 2010, the European Committee on Antimicrobial Susceptibility Testing (EUCAST) changed the MIC cutoff points used to classify the level of resistance. Currently, according to the latest EUCAST data, the breakpoints for sensitive and resistant *S. aureus* strains overlap, and the intermediate range (reduced sensitivity) has been excluded as a criterion (Table 1).

**Table 1:** Criteria for vancomycin resistance in *Staphylococcus aureus* ; EUCAST (European Committee on Antimicrobial Susceptibility Testing) and CLSI (Clinical and Laboratory Standards Institute) guidelines in the years 2009–2022 are presented

Year	EUCAST			CLSI		
	vancomycin MIC [mg/l]			vancomycin MIC [mg/l]		
	sensitive	reduced sensitivity	resistant	sensitive	reduced sensitivity	resistant
2009	≤ 4	8	16	≤ 2	4–8	≥ 16
2010	≤ 1	2	≥ 4	≤ 2	4–8	≥ 16
2011	≤ 2	–	≥ 4	–	–	–
2013	≤ 2	–	≥ 4	–	–	–
2022	≤ 2	–	> 2	≤ 2	4–8	≥ 16

Moreover, according to the report by Ghahremani et al. (2018), a single strain can be phenotypically defined as VISA (intermediate susceptibility to vancomycin) and, based on molecular analysis, as VRSA (resistant to vancomycin). These reports raise serious concerns regarding control measures and the reliability of laboratory tests for screening for resistant strains.

Shortly after the discovery of the Mu50 strain, it was initially believed that its resistance to vancomycin could be attributed to the exceptionally thick cell wall (about 40 layers of peptidoglycan), which is twice as thick as in typical MRSA strains, and its ability to synthesize several times more murein precursors (Hiramatsu et al., 1997). However, further research revealed that these features alone are not sufficient to cause resistance, and the key changes occur at earlier stages of cell wall biosynthesis. Genomic analyses indicated that the areas of change, consisting mainly of single nucleotide substitutions, concern cellular transport, carbohydrate metabolism, and regulatory mechanisms (Kuroda et al., 2001).

Differences also occur in genes related to the tricarboxylic acid metabolism cycle: *ascA*, *pykA*, and *IctE*, which lead to the formation of pyruvate, a key component in the synthesis of GlcNAc-β-(1,4)-MurNAc-pentapeptide – a building block of the cell wall (Kato et al., 2010; Ohta et al., 2004). The increased synthesis of peptidoglycan precursors and transport results in the formation of a cell wall with an unusual structure. A characteristic feature of the Mu50 strain is the presence of glutamate molecules deprived of the amino residue in the murein pentapeptide, which consequently prevents the transpeptidase from producing a peptide bond between L-Lysine and the Glycine carboxyl group of the pentaglycine chain. This modification generates peptidoglycan with a changed spatial structure.

As a result, an increased amount of unbound D-Alanine-D-Alanine (D-Ala-D-Ala) dimers at the ends of pentapeptides and reduced cross-linking are observed (Hanaki et al., 1998; Wang et al., 2022). Free dimers are not incorporated into the peptidoglycan structure but diffuse in the three-dimensional space of the peptidoglycan network, thus constituting a trap for vancomycin before it reaches the proper site of action. Reduced cross-linking allows this glycopeptide to penetrate and capture antibiotic molecules before they reach the site of heterodimer polymerization (GlcNAc-β-(1,4)-MurNAc-pentapeptide), which occurs on the outer surface of the cell membrane. Additionally, trapped vancomycin molecules, considered large among all antibiotics, create steric hindrance for subsequent molecules that can penetrate deep into the wall (Wang et al., 2022). Another feature associated with an increased amount of free D-Ala-D-Ala dimers and vancomycin resistance is a decreased activity of the penicillin-binding protein PBP4, an acyl-serine transferase participating in the polymerization process of this dipeptide.

Autolysis is the self-digestion of the cell wall by peptidoglycan hydrolases called autolysins. In most organisms, autolysis represents programmed cell death. However, in Staphylococci, it also plays a role in antibiotic resistance. For example, VISA strains show reduced autolytic activity, which translates into a less negative net surface charge compared to other strains, and, as a consequence, reduced sensitivity to cationic antimicrobial peptides. Observations made on strains growing on media with vancomycin at a concentration corresponding to a subinhibitory dose reveal that cells grow as amorphous clusters or racemes. More detailed analyses show that they have an unseparated, amorphous cell wall (McAleese et al., 2006).

Simultaneously, a reduced concentration of antibiotic is observed near the grown colonies, confirming previous results indicating the incorporation of vancomycin into the peptidoglycan structure (Xu et al., 2018).

Globally, changes in the level of mRNA expression in vancomycin-susceptible *S. aureus* (VSSA) and vancomycin-resistant VISA strains obtained from the same patient at different stages of long-term vancomycin therapy reveal that the development of the VSSA phenotype is a polygenic and extremely complex process. Comparison of the vancomycin-sensitive variant with the intermediate-susceptible one reveals differences in the expression of at least 224 genes. The observed reduction in the level of mRNA synthesis concerns 169 genes, mainly related to basic metabolism, surface protein synthesis, and the production of toxins and enzymes. Increased expression is indicated for 55 genes mainly related to cellular transport, carbohydrate metabolism, cell wall biosynthesis, and global regulatory processes. The analysis also identifies two genes whose expression is 84-fold and 23-fold higher, respectively, in the case of the VISA variant compared to the susceptible variant, but the functions of these genes have not yet been fully understood (Chang et al., 2003).

Six years after the discovery of the Mu50 strain, the first MRSA strain was isolated in Michigan, USA, showing resistance to vancomycin and glycopeptides due to acquiring the vanA gene cluster from the *Enterococcus* genus (Garrdete and Tomasz, 2014). Gene expression in the *vanA* cluster, located in the transposon Tn1546, leads to the modified synthesis of peptidoglycan precursors, which are the drug target molecules. As a result, instead of binding to D-Alanine-D-Alanine, vancomycin binds with reduced affinity to D-Alanine-D-Lactate or D-Alanine-D-Serine (Park et al., 2024). These observations indicate that glycopeptide resistance can be achieved in two effective ways. Therefore, MRSA has gained worldwide fame as the hospital superbug.

## Mechanisms of antibiotic resistance in MRSA

Protein biosynthesis is the most energy-consuming cellular process. Translational suppression during environmental stress is a universal adaptive mechanism used by all living organisms to reduce energy expenditure. Bacterial ribosome hibernation has emerged as one of the pivotal cellular processes to accomplish translational suppression.

In a limited number of bacteria, including *S. aureus*, a phenomenon seen during the logarithmic growth phase is the presence of 100S ribosome complexes. These 100S ribosomes, also known as hibernating ribosomes, consist of two 70S monomers connected by a small 30S subunit, and their formation involves HPFSa – a factor promoting hibernation (Kato et al., 2010; Ueta et al., 2010).

Typically, in most bacteria, the presence of 100S complexes is associated with entering the stationary phase, when growth slows down and stress factors appear. The 100S ribosomes constitute a reservoir of ribosomes capable of immediate dissociation and resumption of translation when favorable conditions arise again. Research indicates that the viability of bacteria deprived of the ability to create hibernating ribosomes by deletion of the HPF factor is limited (Yoshida and Wada, 2014). Based on this, it can be concluded that the formation of hibernating ribosomes is an important survival strategy for bacteria, enabling an effective response to stress.

Observable spontaneous mutations in the laboratory can occur at a frequency of 10^-6^ to 10^-8^ per single cell. This process of mutation and selection has led to the creation of a population of antibiotic-resistant bacteria (Pray, 2008). Additional perspective on the evolution of resistant bacteria has been gained by characterizing horizontal gene transfer (HGT) and extra-chromosomal DNA elements. It is now believed that most cases of antimicrobial resistance result from the acquisition of additional genetic elements, including plasmids, mobile genetic elements (such as insertion sequences and transposons), and genomic islands. These elements already contain integrated antibiotic resistance genes and are transmitted via HGT not only within one species but also between separate species, even from different genera.

All types of horizontal gene transfer, including transduction, transformation, and conjugation, have been observed in the laboratory of *S. aureus* (Mehta et al., 2023). The specific contribution of each to the natural environment remains uncertain. Plasmids are a crucial genetic element in acquiring antibiotic resistance genes. The insertion sequences within them enable complete or partial recombination, serving as vectors for mobile genetic elements containing resistance genes. These elements enable transfer both between plasmids and from a plasmid to a site in the chromosome without the use of genetic homology. The accumulation of many resistance genes on one plasmid or their presence within a transposon on the chromosome explains the emergence of multidrug-resistant Staphylococci (Saunders, 1984; Al-Trad et al., 2023).

An additional observation common to bacteria more likely to be resistant to antibiotics is a genome size of 2.5 Mb or more (MacLean and San Millan, 2019). In the case of *S. aureus*, the genome size is as much as 2.8 Mb. This has led to the suggestion that the ability of a bacterium to evolve toward a multidrug-resistant phenotype may be a function of genome size (Projan, 2007). According to this hypothesis, organisms with larger genomes have a greater variety of additional genetic elements and a greater ability to make compensatory mutations that increase the stability of insertions, transpositions, and recombinations in the genome. If successful, such compensatory mutations block reversion to the susceptible phenotype even in the absence of selection, contributing to the maintenance of resistant strains (Jalasvuori and Penttinen, 2017).

One of the most characteristic mobile genetic elements in MRSA transferred during HGT is the SCC*mec* – Staphylococcal Cassette Chromosome *mec* (Fang et al., 2024). It is the main carrier of the β-lactam antibiotic resistance genes *mec* (*mecA*, *mecB*, *mecC* ) along with the genes controlling their expression (*mecR1* and *mecI* ). *SCCmec* is located near the origin of chromosome replication at the *attB* insertion site and consists of three basic elements.

The first element is the *ccr* gene complex consisting of *ccrAB* and/or *ccrC*, surrounded by an open reading frame (ORF). The second element is the *mec* complex, composed of the *mec* genes, the inertial sequence, and surrounding ORFs. The third element is called the J region.

The *ccr* complex is crucial for multidrug resistance, enabling the insertion of additional resistance genes thanks to the specific recombination sites *ccrAB* and *ccrC*, which are highly recognizable among all strains of the *Staphylococcus* genus. *SCCmec* acts as a chromosomal reservoir for the insertion of additional antibiotic resistance determinants along with transposable elements. For example, MRSA can acquire Tn554, which encodes resistance to MLS (macrolides, lincosamides, streptogramins) antibiotics: erythromycin A, erm(A), and spectinomycin (spc) (Murphy et al., 1985). This represents an efficient exchange of genetic information enabling quick adaptation to environmental pressures related to antibiotics and contamination with heavy metals such as cadmium. The mobile genetic element *SCCmec* is reported to be crucial in the evolution of MRSA in terms of acquiring resistance genes (Liu et al., 2016).

The identified mechanisms of antibiotic resistance in MRSA represent diverse models of antibacterial abolition. One mechanism involves the production of specific enzymes that inactivate the antibiotic through enzymatic degradation, involving hydrolysis of the antibiotic's functional group (De Pascale and Wright, 2010). *S. aureus* MRSA uses this mechanism against β-lactam antibiotics, including penicillin. The extracellular β-lactamase produced deactivates the antibiotic by hydrolyzing the β-lactam ring. The β-lactamase gene *blaZ* is found in transposons carried by a diverse group of so-called heavy metal and β-lactam resistance plasmids. This type of resistance is also observed towards chloramphenicol. Antibiotic detoxification occurs with the participation of the inducible enzyme chloramphenicol acetyltransferase, which is a product of the *cat* gene located on the pC221 plasmid (Berg et al., 1998).

Another molecular pattern is based on a change in the structure of the antibiotic’s target protein. This mechanism is activated, for example, against antibiotics from the group of semisynthetic penicillins and cephalosporins against which β-lactamase activity is ineffective. The protein product of the *mec* genes located in the *SCCmec* encodes the PBP2a protein, which has a reduced affinity for the antibiotic (Bilyk et al., 2023; Fergestad et al., 2020).

The model of preventing drug action by structural changes in the target protein also represents fusidic acid resistance. Chromosomal mutations in *fusA*, the gene encoding the elongation factor EF-G, an essential translation factor required for peptide translocation and ribosome recycling, generate modifications that block antibiotic binding. Resistance to fusidic acid is also demonstrated by efflux pump activity, which removes xenobiotics by pumping them out of the cell (Chen et al., 2011).

Active transport of antibiotics plays a significant role in multidrug resistance. Genes involved in the active transport of xenobiotics out of the cell are widely identified in multidrug resistance plasmids such as *pSK1* (Jensen et al., 2010). This mechanism may exist independently, but in the vast majority of cases, it has a synergistic effect with other resistance mechanisms (Nazaro, 2022). The presence of efflux pumps predisposes bacteria to develop antibiotic resistance.

An example of assisted evolution by efflux pumps is ciprofloxacin resistance in *S. aureus* (Papkou et al., 2020). In a study involving 222 isolates cultured in the presence of the antibiotic, resistance developed in those with increased expression of *norA*, one of the most studied efflux pump genes in staphylococci. Overexpression of *norA* led to higher spontaneous mutation rates among genes encoding topoisomerase IV and DNA gyrase, which are targets for fluoroquinolone antibiotics (Papkou et al., 2020). Such mutations (*Ser80Phe* and *Glu84Lys* ) disrupt the water-metal ion bridge between quinolones and topoisomerase IV and therefore may be responsible for tolerant strains developing resistance to ciprofloxacin.

The opposite mechanism of reducing antibiotic penetration is preventing biofilm formation. Several genes have been identified to be involved in biofilm formation in a highly biofilm-forming clinical *S. aureus* isolate. One of the characterized genes, *bfd2*, encoding a hypothetical protein, shows all the features of an efflux pump belonging to the MFS (major facilitator superfamily), one of the five superfamilies of efflux pumps associated with multidrug resistance. Studies have also shown that MgrA, a pleiotropic regulator in *S. aureus*, acts as a negative regulator of the NorB and NorC135 efflux pumps and at the same time inhibits biofilm formation (Liu et al., 2010). Subsequent studies demonstrating the relationship between these resistance mechanisms reported that the relative expression levels of the MFS family efflux pump genes *mdeA*, *norB*, and *norC* were increased during biofilm formation. The *NorB* and *NorC* efflux pumps can export cetrimide, ethidium bromide, and quinolones (Truong-Bolduc et al., 2006).

The successful development of antibiotic resistance is most often the result of the synergistic effect of interlocking biochemical pathways (Darby et al., 2023). For example, fluoroquinolone resistance can occur due to three different biochemical routes, all of which may coexist in *S. aureus* at a given time, producing an additive effect: 1) mutations in genes encoding the target site of FQs (DNA gyrase and topoisomerase IV), 2) over-expression of efflux pumps that extrude the drug from the cell, and 3) protection of the FQ target site by a protein designated Qnr. Selected documented resistance determinants are presented in Table 2. This list highlights the diversity of resistance mechanisms in the case of *S. aureus* bacteria.

**Table 2:** List of selected genetic determinants of antibiotic resistance in *Staphylococcus aureus*

Antibiotic	Determinant of resistance	Mechanism of resistance	Localization	Genetic element
Biocides	*qacA* *qacB* *qacC*	multidrug efflux pumps	plasmide	pSK1 pSK23 pSK41
Bleomycin	*Ble*	bleomycin-binding protein	plasmide chromosome	pUB110
Chloramphenicol	*cat*	chloramphenicol acetyltransferase	plasmide	pC221
	*cfr*	23S rRNA methyltransferase	chromosome	IS*21-558*
Fluoroquinolones	*grlA/B*	DNA topoisomerase IV	chromosome	–
	*gyrA/B*	DNA gyrase		
	*norA*	multidrug efflux pumps		
Fusidic acid	*fusA*	elongation factor EF-G	chromosome	–
	*fusB*	detoxification	plasmide	Pub101
Gentamicin Kanamicin	*aacA-aphD*	6′-aminoglicoside N-acetylotransferase 2″-aminoglicoside phosphotransferase	plasmide :: transposone chromosome :: transposone	Tn*4001*
Linezolide	23S rRNA genes	23S rRNA	chromosome	–
Methicilin Oxacilin	*mecA*	PBP2a	chromosome :: SCC	SCC*mec*
MLS	*erm(A)*	rRNA N-6-adenine methyltransferase	chromosome :: transposone	Tn*554*
	*erm(B)*		plasmide :: transposone	Tn*551*
	*erm(C)*		plasmide	pE194
Streptogramin A	*vga(A)*	ABC efflux pumps	plasmide	pIP524
	*vga(B)*			pIP1633
	*vat(A)*	virginiamicin acetyltransferase		pIP680
	*vat(B)*			pIP524
Streptogramin B	*vgb(A)*	streptogramin B lyase	plasmide	pIP524
Mupirocin	*ileS*	isoleucyl-tRNA synthase	plasmide	pIP524
	*ileS-2*	isoleucyl-tRNA synthase	chromosome	–
Neomycin	*aphA-3*	aminoglycoside phosphotransferase	chromosome :: transposone	Tn*5404*
Kanamycin	*aadD*	aminoglycoside phosphotransferase	plasmide	pUB110
Penicillin	*blaZ*	β-lactamase	plasmide :: transposone plasmide :: chromosome	Tn*552*
	*mecA*	PBP2a	chromosome :: SCC	SCC*mec*
Ryphampcin	*rpoB*	RNA polimerase β	chromosome	–
Spectinomycin	*spc*	spectinomycin adenylotransferase	chromosome :: transposone	–
Streptomycin	*str*	spectinomycin adenylotransferase	plasmide	pS194
	*sat4*	streptomycin adenylotransferase	chromosome :: transposone	Tn*5405*
Tetraciclin	*tetA(K)*	efflux pump	plasmide chromosome :: plasmide	pT181
	*tetA(L)*		plasmide	pKKS2187
	*tetA(M)*	ribosome protection protein	chromosome :: transposone	Tn*5801*
Trimetoprim	*dfrA*	dihydrofolic reductase	plasmide	pSK639
	*dfrB*		chromosome	–
Vancomycin Teicoplanin	*vanHAXYZ*	glicopeptides resistance	plasmide :: transposone	Tn1546

## Perspectives

One of the most common drug-resistant microorganisms monitored by WHO is the methicillin-resistant *S. aureus* (MRSA) strain. This Gram-positive bacterium is one of the main agents associated with nosocomial infections. For many years, methicillin, a penicillin-resistant to β-lactamases, was considered the basic drug against staphylococcal infections. This antibiotic works by blocking penicillin-binding proteins, which are involved in the synthesis of peptidoglycan, a key component of the cell wall. *S. aureus* usually acquires resistance through the acquisition of the *mecA* gene, which encodes the penicillin-binding protein PBP-2a. Although semisynthetic penicillins, such as methicillin, flucloxacillin, dicloxacillin, or nafcillin, can inhibit the action of other PBPs normally found in *S. aureus* (Tuon et al., 2023), the methicillin-resistant PBP-2a protein complements their action, allowing the bacteria to grow in the presence of these antibiotics. For this reason, the use of penicillins and semisynthetic penicillins is limited. Additionally, many *S. aureus* strains have acquired resistance to other commonly used groups of antibiotics through spontaneous mutation or horizontal gene transfer, including 1) amoxicillin, ampicillin, benzylpenicillin, 2) fluoroquinolones, and tetracyclines, and 3) gentamicin, tobramycin, netilmicin, and amikacin (Urban-Chmiel et al., 2022).

To address the challenge of antibiotic resistance, new antibiotics have been developed to target MRSA, including vancomycin, daptomycin, and linezolid. However, despite efforts to prescribe these drugs selectively to reduce the risk of resistance, MRSA has developed mechanisms to resist them as well (Esposito et al., 2023). As a result, vancomycin once considered the drug of “last resort”, is no longer effective against many MRSA isolates. This reality has spurred the search for new methods of treating drug-resistant infections through alternative therapies and a deeper understanding of the mechanisms underlying antibiotic resistance.

A notable trend in this area is the modification of existing bactericidal compounds rather than the design of entirely new antibiotics. With new bacterial targets limited, researchers are exploring ways to modify the structure of vancomycin to bypass resistance mechanisms while maintaining its bactericidal effects. Many strategies focus on introducing cationic charges and lipophilic elements into the vancomycin structure to target the bacterial cell membrane (Blaskovich et al., 2018; Yarlagadda et al., 2014). Some of these modifications have shown promise in restoring vancomycin’s efficacy against infections, but further optimization is needed to prevent the emergence of new resistance mechanisms (Esposito et al., 2023).

Since MRSA infections are associated with biofilm formation, novel strategies based on the destruction of the extracellular matrix, which constitutes the bacterial biofilm, are being developed using nanomedicine. In 2019, Khalid et al. reported that rhamnolipid-coated silver and iron oxide nanoparticles are effective in eradicating *S. aureus* (Khalid et al., 2019). Such nanoparticles might encapsulate antimicrobial drugs; for example, positively charged nanoparticles containing rifampicin showed lower MIC values compared to nonencapsulated rifampicin by inhibiting the growth of *S. aureus* (Tran et al., 2018). Other scientists have explored alternative strategies to overcome MRSA antibiotic resistance using plant-derived substances. Examples of successful inhibition of MRSA growth include the usage of alkaloids, which inhibit ATP synthase (Mun et al., 2014), or coumarin, which inhibits DNA gyrase (Bazzaz et al., 2010).

There are five ongoing clinical trials registered in the ClinicalTrials.gov database aimed at MRSA treatment. Three of them are testing new antimicrobial agents. The first trial (study ID: NCT04104178) evaluated oral antibiotic clindamycin for MRSA throat carriers. The second trial (study ID: NCT05225558) is investigating delpazolid, a novel oxazolidinone with cyclic amidrazone, combined with vancomycin in hospitalized adults with MRSA bacteremia. The third trial (study ID: NCT03637400) is studying the efficacy of doxycycline (tetracycline class) and trimethoprim/sulfamethoxazole (one part trimethoprim – dihydrofolate reductase inhibitor, and five parts sulfamethoxazole – sulfonamide class) for curing uncomplicated skin and soft tissue infection.

Additionally, two clinical trials are focused on optimizing vancomycin dosing. The first trial (study ID: NCT04793152) aims to compare intravenous vancomycin dosing strategies targeting a trough level of 10 to 15 mg/l versus AUC of 400–600 assuming a MIC of 1 μg/ml by broth microdilution for serious MRSA infections. The second trial (study ID: NCT00945152) is investigating the usage of Vancogel™, a complex gel formulation with 1.25–1.50% vancomycin, for MRSA elimination from open wounds. Despite continuous discoveries of various alternative strategies to combat antimicrobial resistance, none of them are currently being successfully used in vancomycin-resistant *S. aureus*.
